# Interleukin‐18 expression in oral squamous cell carcinoma: its role in tumor cell migration and invasion, and growth of tumor cell xenografts

**DOI:** 10.1002/2211-5463.12532

**Published:** 2018-10-31

**Authors:** Yuyang Li, Zhiming Xu, Jia Li, Shuofeng Ban, Congcong Duan, Weiwei Liu

**Affiliations:** ^1^ Department of Dental Implantology School and Hospital of Stomatology Jilin University Jilin Provincial Key Laboratory of Tooth Development and Bone Remodeling Changchun Jilin China; ^2^ Department of Oral and Maxillofacial Surgery School and Hospital of Stomatology Jilin University Jilin Provincial Key Laboratory of Tooth Development and Bone Remodeling Changchun Jilin China

**Keywords:** growth, IL‐18, interleukin‐18, migration and invasion, oral squamous cell carcinoma, xenograft model

## Abstract

Oral squamous cell carcinoma (OSCC) is one of the most common head and neck malignancies. Advanced stages of the disease are associated with poor survival, highlighting a need for new treatment modalities. We previously showed that the proinflammatory cytokine interleukin‐18 (IL‐18) has a tumor suppressive role in OSCC. Here, we investigated the effects of IL‐18 on proliferation, migration, and invasion of OSCC cells *ex vivo* and *in vitro*, and in nude mouse xenografts. We report that expression of tankyrase 2 (TNKS2), β‐catenin, and N‐cadherin was higher in tumor cells than in normal mucosae, whereas the expression of IL‐18 and E‐cadherin was higher in normal than in tumor tissues. Elevated expression of IL‐18 (*P *<* *0.01) and E‐cadherin (*P *=* *0.034) was associated with tumor differentiation, whereas expression of TNKS2 (*P *<* *0.01), β‐catenin (*P* = 0.012), and N‐cadherin (*P* < 0.01) was associated with tumor de‐differentiation. Furthermore, compared with the vector control, IL‐18 overexpression promoted tumor cell migration and invasion (*P *<* *0.01), but inhibited growth of tumor cell xenografts (*P *<* *0.05). At the protein level, expression levels of IL‐18 (*P *<* *0.01), TNKS2 (*P* = 0.045), β‐catenin (*P* = 0.028), and N‐cadherin (*P* = 0.068) were upregulated in tumor cells after IL‐18 overexpression compared with those of the vector control mice, whereas expression levels of E‐cadherin (*P* = 0.045) were decreased. In conclusion, our data suggest that IL‐18 overexpression induces oral SCC cell invasion and metastasis by promoting the tumor cell epithelial–mesenchymal transition via the Wnt/β‐catenin signaling pathway.

**Abbreviations**
EMT: epithelial–mesenchymal transition
IL‐18: interleukin‐18
IOD: integrated optical density
MTT: 3‐(4,5‐dimethylthiazol‐2‐yl)‐2,5‐diphenyl‐tetrazolium bromide
OSCC: oral squamous cell carcinoma
TNKS2: tankyrase 2
TSCC: tongue squamous cell carcinoma

Oral squamous cell carcinoma (OSCC) is one of the most common head and neck malignancies. It occurs in the oral cavity—including the lip, tongue, mouth, gingiva, and palate 1—and accounts for more than 95% of all oral cancers 2, 3. OSCC exhibits tumor cell infiltrating growth as well as high malignance, invasion, and metastasis 2, 3, 4. Approximately 75% of oral cancer is linked to tobacco smoke and excessive alcohol consumption, while poor oral hygiene, irritation (induced by poorly fitting dentures), poor nutrition, and chronic infections in the oral cavity are also risk factors in development of oral cancer 5. Early detection of oral cancer results in effective treatment options and a favorable prognosis, while advanced stages of the disease are associated with poor survival of patients 6. Thus, research and development of novel therapy (such as molecular gene targeting therapy or immunotherapy) could improve the efficacy of treatment and prolong patient survival 7 .

Interleukin‐18 (IL‐18, also known as interferon‐gamma inducing factor) is a proinflammatory cytokine that possesses many different biological functions, such as regulation of T‐cell‐mediated immunoreaction and promotion of other cytokine secretion, and is secreted mainly by monocytes and activated macrophages. Previous studies also showed that IL‐18 possesses an antitumor activity in various human cancers 8, 9, 10, 11; for example, treatment of human cancer patients with intravenous injection of recombinant human IL‐18 (rhIL‐18) showed antitumor effects 10 and the combination with other cytokines could also achieve tumor cell killing and tumor inhibitory effects 8, 12.

In our previous studies, we demonstrated that IL‐18 had a tumor suppressive function in oral squamous cell carcinoma cells 9, 11, 12. However, other studies showed that IL‐18 was able to promote tumor development and progression by showing higher levels of serum IL‐18 in patients with gastric cancer 13 or oral cancer 14. Jurecekova *et al*. 15 assessed the level of IL‐18 in prostatic cancer and discovered that IL‐18 expression was associated with advanced grades of tumor and inversely with tumor prognosis and invasion. Furthermore, the epithelial–mesenchymal transition (EMT) denotes the change in which epithelial cells lose their cell polarity and cell–cell adhesion capacity but gain migratory and invasive properties, similar to mesenchymal cells 16, 17, and is one of the main characteristics of tumor cell migration and invasion 16, 18, 19. At the protein level, E‐cadherin (a member of calcium‐dependent cellular adhesion family) maintains cell polarization and connection between epithelial cells 20, 21. Previous studies showed that the level of E‐cadherin expression was reduced in epithelial cells when such epithelial cells underwent the EMT 20, 21 but another cadherin (N‐cadherin) was upregulated during promotion of mesenchymal cell properties 22, 23, resulting in increase in cancer cell migration and invasion 16, 18, 19, 22, 23. Thus, the ratio of E‐cadherin to N‐cadherin (E/N) was used as an indicator of the EMT 24, 25.

In addition, previous studies demonstrated that the Wnt/β‐catenin signaling pathway also participated during cancer cell EMT 26, 27, accompanied by downregulated epithelial markers, E‐cadherin and keratin, and upregulated mesenchymal markers such as N‐cadherin and vimentin, while tankyrase 2 (TNKS2) plays an important role in tumor cell migration and invasion 28 and TNKS2 inhibition was able to downregulate activity of the Wnt/β‐catenin signaling pathway and thereby reduce tumor cell growth 29. Overall, tumor development and progression involve multiple gene alterations and changes in the host immune defense; increased cell proliferation, loss of apoptosis, and tissue invasion and metastasis could all result in tumorigenesis and progression 30. IL‐18 plays a role in regulation of cell functions in these areas of the human body.

Thus, based on findings from previous studies, our research team collected OSCC tissue samples to analyze expression of different proteins and then investigated the effect of IL‐18 overexpression on regulation of tumor cell growth, migration, and invasion capacity *in vitro* as well as tumor cell xenograft growth in a nude mouse model. We expected to provide useful information regarding the role of IL‐18 in OSCC.

## Materials and methods

### Tissue samples

In this study, we obtained 38 paraffin‐embedded OSCC and six adjacent normal tissue samples (histopathologically diagnosed as OSCC) from the Department of Oral Pathology, Jilin University (Changchun, China). The patients were under medical care between 2014 and 2017, and no patients received radiotherapy or chemotherapy before surgery. This study was approved by the Ethics Committee of Hospital of Stomatology, Jilin University, and undertaken with each patient understanding and giving written informed consent before participation. The study methodologies conformed to the standards set by the Declaration of Helsinki. The medical records of these patients were obtained to extract detailed clinicopathological data (including age, gender, and tumor histological grade, pathological stage, and lymph node metastasis). The paraffin‐embedded tissue blocks were sectioned into 4‐μm‐thick sections for hematoxylin and eosin staining for OSCC confirmation and immunohistochemistry.

### Immunohistochemistry

Tissue sections from human OSCC and adjacent normal samples as well as mouse tumor xenografts were subjected to immunohistochemical analysis using an immunohistochemical kit (MXB Biotechnologies, Fuzhou, China). The polyclonal antibodies against IL‐18, β‐catenin, E‐cadherin, and N‐cadherin were obtained from ABclonal Biotech Co., Ltd (Wuhan, China), and TNKS2 was obtained from Beijing Biosynthesis Biotechnology Co., Ltd (Beijing, China). Dilution of each antibody for human tissue sections was at 1 : 100, and anti‐IL‐18, β‐catenin, E‐cadherin, and N‐cadherin antibodies for mice were diluted at 1 : 150 and TNKS2 at 1 : 100. In brief, the sections were deparaffinized in xylene and rehydrated in graded ethanol solutions and tap water. Next, the sections were subjected to microwave‐induced antigen retrieval in a citric acid buffer (10 g·L^−1^, pH 6.0; Boster Biological Technology, Ltd, Wuhan, China) for 10 min and then incubated in 3% H_2_O_2_ diluted in PBS to block potential endogenous peroxidase activity for 10 min. Next, the sections were blocked with normal sheep serum in PBS at a dilution of 1 : 4 at room temperature for 20 min and then with the primary antibody diluted in PBS at 4 °C overnight.

The sections were washed three times in PBS on the following day and then incubated with biotin‐labeled sheep anti‐rabbit IgG for 10 min and washed with PBS for 10 min with peroxidase‐labeled streptavidin labeling. After being washed with PBS, the sections were subjected to a color reaction using 3,3′‐diaminobenzidine solution (MXB Biotechnologies) for 3 min and counterstaining with hematoxylin. Finally, the sections were dehydrated in graded ethanol solutions, cleared in xylene and mounted under coverslips. The negative control sections were incubated with PBS instead of the primary antibody.

The immunostained sections were reviewed and scored under a light microscope by two investigators independently in a blinded fashion. A minimum of five microscopic fields at a magnification of ×400 was randomly selected, and each section was scored according to the degree of clearly visible yellow or brown precipitation for positive immunoreaction. Scoring of antibody immunoreactions in each patient sample involved evaluating the percentage of positive staining and the staining intensity—i.e. percentage of positivity was defined as 0 (negative, no visible reaction or less than 10% of staining), 1 (10–25% of cells stained), 2 (25–50% of cells stained), 3 (50–75% of cells stained), and 4 (>75% of cells stained). The staining intensity was evaluated semi‐quantitatively using a four‐level system (0, negative; 1, weak; 2, moderate; and 3, strong) as described by da Silva *et al*. 31. The staining index for each section was then reached using the formula: index value = score of percentage of positivity × the staining intensity score. For statistical analysis, the samples were categorized into two groups, i.e. negative (≤5 staining index score) and positive (>5 staining index score), as described in the study by da Silva *et al*. 32. For mouse xenograft samples, the immunostaining data were quantified for integrated optical density (IOD) using image‐pro plus software (Media Cybernetics, Sarasota, FL, USA).

### Cell line and culture

A tongue squamous cell carcinoma (TSCC) cell line, CRL1623, was purchased from American Type Culture Collection (Manassas, VA, USA) and cultured in Dulbecco's modified Eagle's medium/F12 (Invitrogen, Carlsbad, CA, USA) at a ratio of 1 : 1 supplemented with 10% fetal bovine serum (PAA Laboratories, Pasching, Austria), 12 g·L^−1^ sodium bicarbonate (Sigma‐Aldrich, St Louis, MO, USA), 2.5 mmol·L^−1^ glutamine (Invitrogen), 15 mmol·L^−1^ HEPES (Sigma‐Aldrich), 0.5 mmol·L^−1^ pyruvic acid sodium (Sigma‐Aldrich), 400 μg·L^−1^ hydrocortisone (Sigma‐Aldrich), 100 U·mL^−1^ penicillin, and 100 mg·L^−1^ streptomycin at 37 °C in a humidified incubator with 5% CO_2_. The medium was refreshed every 3 days, and cells were passaged with 0.25% trypsin (Invitrogen) and 0.03% EDTA (Invitrogen). All experiments were carried out using cell growth in the logarithmic phase.

### Establishment of stable IL‐18‐expressed CRL1623 sublines

Construction of eukaryotic expression vector pcDNA3.1(+)‐IL‐18 and cell viability 3‐(4,5‐dimethylthiazol‐2‐yl)‐2,5‐diphenyl‐tetrazolium bromide (MTT) assay was carried out as described in our previous study 33. In this study, CRL1623 cells were grown and stably transfected with pcDNA3.1(+)‐IL‐18 and pcDNA3.1, respectively, using Lipofectamine 2000 reagent (Invitrogen) and then stabilized in G418 (Invitrogen)‐containing medium using single cell cloning and expansion. The stable sublines were then maintained at a G418‐reinforced minimal lethal dose (650 μg·μL^−1^) and labeled as CRL1623‐IL‐18 and CRL1623‐vec, respectively.

### Transwell^®^ assay

Stable CRL1623 sublines were grown in the full cell culture medium containing G418 and re‐seeded into the upper chamber (400 μL for 10 000 cells per chamber), while the bottom chambers were filled with 600 μL of the growth medium containing 10% fetal bovine serum. The CRL1623 cells were cultured for 24 or 48 h. For the tumor invasion assay, the filter of the Transwell chamber was precoated with 50 μL of Matrigel (BD Biosciences, San Jose, CA, USA) at a dilution of 1 : 3 with the culture medium. At the end of the experiments, cells remaining on the surface of the upper chambers were removed using a cotton swab, while the cells that had migrated or invaded into the lower surface of the Transwell chambers were fixed in 10% formalin and subsequently stained with 1% crystal violet for 15 min. The filters were then reviewed and the numbers of cells in five randomly selected fields were counted under a microscope. The experiments were carried out using five parallel Transwell chambers for each group.

### Nude mouse xenograft model

The animal experiment was approved by the Institutional Animal Care and Use Committee (IACUC) of Jilin University. In brief, 12 BALB/c‐nu mice aged 5–6 weeks and weighing 18–22 g were obtained from HuaFuKang Bioscience Co. Inc. (Beijing, China) and housed under controlled temperature and humidity in alternating 12‐h light and dark cycles in a specific pathogen‐free condition. The mice received specific pathogen‐free mouse chow and were allowed sterile water *ad libitum*. The mice were inoculated with 200 μL of CRL1623‐IL‐18 or CRL1623‐vec cells, respectively, at the left front limb. The cells were in logarithmic growth phase and detached from the cell culture dishes, and the cell suspension was adjusted to 1 × 10^7^·mL^−1^ with PBS. After injection, the xenograft formation and growth were monitored with a Vernier caliper every 3 days for the major axis *a* (mm) and minor axis *b* (mm) of xenografts and the volume of the xenografts was calculated using the formula *V* = &frac12;*ab* ^2^ 34. Thirty days later, the mice were sacrificed and tumor xenografts were resected and then fixed in 10% formalin solution, dehydrated in graded alcohol and *n*‐butanol, embedded in paraffin, and sectioned.

### Statistical analysis

All statistical analyses were performed using spss v18.0 software (SPSS, Chicago, IL, USA). Comparison between groups was assessed using one‐way analysis of variance, Student's *t* test, or the χ^2^ test. Comparison between factors was analyzed using Pearson's correlation test. A *P* value <0.05 was considered statistically significant.

## Results

### Characteristics of OSCC patients

In this study, we recruited 38 OSCC patients—28 males (73.7%) and 10 females (26.3%)—with a mean age of 61.92 years (range 42–81 years). Histologically, 12 cases were well differentiated, 13 were moderately differentiated, and 13 were poorly differentiated OSCCs, while 15 cases (3 + 12, 39.5%) had early clinical stage (I + II) and 23 cases (18 + 5, 60.5%) had advanced stages (III + IV) of OSCC. Furthermore, nine cases (23.7%) had lymphatic metastasis, while 29 cases (76.3%) had no lymphatic metastasis (Table 1).

**Table 1:** Clinicopathological features and prognoses of 38 patients

Variable	Number of patients	%
Gender		
Male	28	73.7
Female	10	26.3
Histological grade		
Well	12	31.6
Moderate	13	34.2
Poor	13	34.2
Clinical stage		
T1	3	7.9
T2	12	31.6
T3	18	47.4
T4	5	13.1
Lymphatic metastasis		
No	29	76.3
Yes	9	23.7

### Association of differential protein expression between OSCC and normal tissue samples

**Figure 1:**
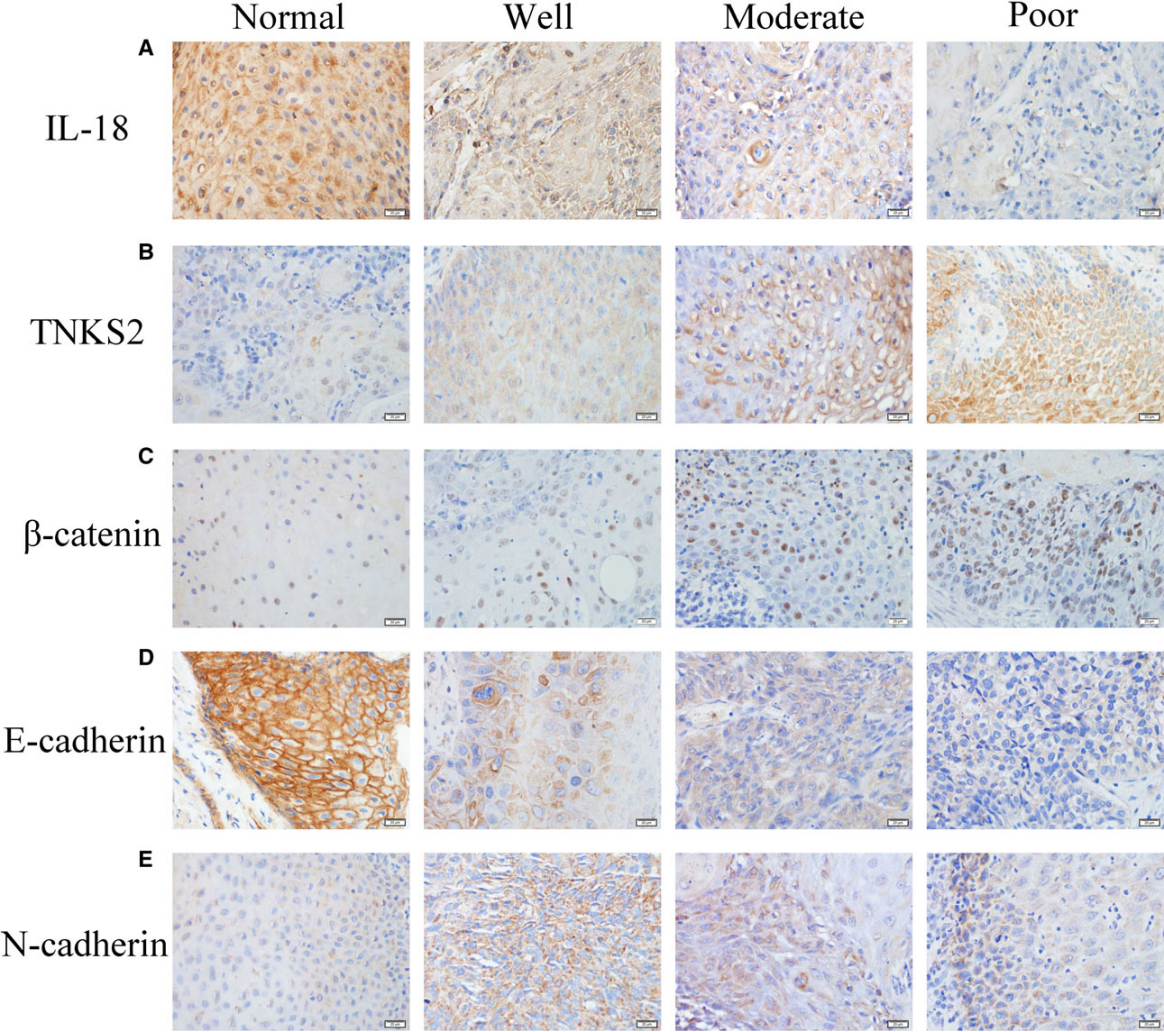
Differential expression of different proteins in OSCC tissues (×400). (A,D) IL‐18 and E‐cadherin expression was reduced in OSCC tissues and associated with OSCC differentiation (*P *< 0.05). (B,C,E) Expression of TNKS2, β‐catenin, and N‐cadherin was upregulated in OSCC tissues and associated with tumor de‐differentiation (*P* < 0.05). Scale bars: 20 µm.

Data on differential protein expression in OSCC tissue samples are shown in Table 2. Specifically, levels of IL‐18 and E‐cadherin expression were higher in normal tissues than in OSCC, whereas β‐catenin, N‐cadherin, and TNKS2 proteins were weaker in normal tissues than in OSCC. The expression of IL‐18 and E‐cadherin was associated with tumor differentiation (*P* < 0.05), whereas the expression of β‐catenin, N‐cadherin, and TNKS2 was associated with tumor de‐differentiation (*P* < 0.05; Fig. 1). Moreover, there was a positive association between TNKS2 *vs* β‐catenin (*P* < 0.01) or N‐cadherin (*P* = 0.02) and between β‐catenin and N‐cadherin (*P* < 0.01), whereas there was an inverse association between TNKS2 and IL‐18 (*P* = 0.06) or E‐cadherin (*P* = 0.06) and between E‐cadherin and β‐catenin (*P* = 0.01) or N‐cadherin (*P *<* *0.01; Table 3).

**Table 2:** Expression of different proteins in OSCC tissues. *P* values determined by Student's *t* test

Variable		IL‐18 (*n* (%))	*P*	β‐Catenin (*n* (%))	*P*	TNKS2 (*n* (%))	*P*	E‐cadherin (*n* (%))	*P*	N‐cadherin (*n* (%))	*P*
Gender	Male	16 (57.1)	0.36	11 (39.3)	0.38	8 (28.6)	0.43	9 (32.1)	0.43	10 (35.7)	0.39
	Female	4 (40)		3 (30)		3 (30)		3 (30.0)		5 (50)	
Property	Carcinoma	20 (51.3)	0.09	14 (35.9)	0.06	11 (28.2)	0.06	12 (30.8)	<0.01	15 (38.5)	0.04
	Normal tissues	5 (83.3)		0 (0)		0 (0)		5 (83.3)		0 (0)	
Histological grade	Well	10 (83.3)	<0.01	2 (16.7)	0.012	1 (8.3)	<0.01	6 (50%)	0.034	1 (8.3)	<0.01
	Moderate/poor	10 (38.5)		12 (46.2)		10 (38.5)		6 (23.1)		14 (38.9)	
Clinical stage	T1 + T2	8 (53.3)	0.35	3 (20)	0.10	2 (13.3)	0.034	6 (40.0)	0.62	4 (26.7)	0.025
	T3 + T4	12 (46.2)		11 (47.8)		9 (39.1)		6 (26.1)		11 (47.8)	
Metastasis	Yes	2 (22.2)	0.056	5 (55.6)	0.068	5 (55.6)	<0.01	2 (22.2)	0.23	7 (77.8)	0.026
	No	18 (62.1)		9 (31.0)		6 (20.7)		10 (34.5)		8 (27.6)	

**Table 3:** Correlations of different protein expressions in OSCC tissues *vs* TSCC xenografts by Pearson's correlation test

Protein comparison	OSCC tissues		TSCC xenografts	
	Correlation index	*P*	Correlation index	*P*
IL‐18 *vs* TNKS2	−0.31	0.06	0.56	0.06
IL‐18 *vs* β‐catenin	−0.23	0.17	0.60	0.04
IL‐18 *vs* E‐cadherin	0.20	0.24	−0.76	<0.01
IL‐18 *vs* N‐cadherin	−0.27	0.11	0.71	0.01
TNKS2 *vs* β‐catenin	0.56	<0.01	0.87	<0.01
TNKS2 *vs* E‐cadherin	−0.52	0.01	−0.41	0.19
TNKS2 *vs* N‐cadherin	0.50	0.02	0.11	0.74
β‐catenin *vs* E‐cadherin	−0.50	0.01	−0.38	0.23
β‐catenin *vs* N‐cadherin	0.52	<0.01	0.35	0.26
E‐cadherin *vs* N‐cadherin	−0.42	<0.01	−0.75	<0.01

### IL‐18 promotion of TSCC cell migration and invasion *in vitro*

**Figure 2:**
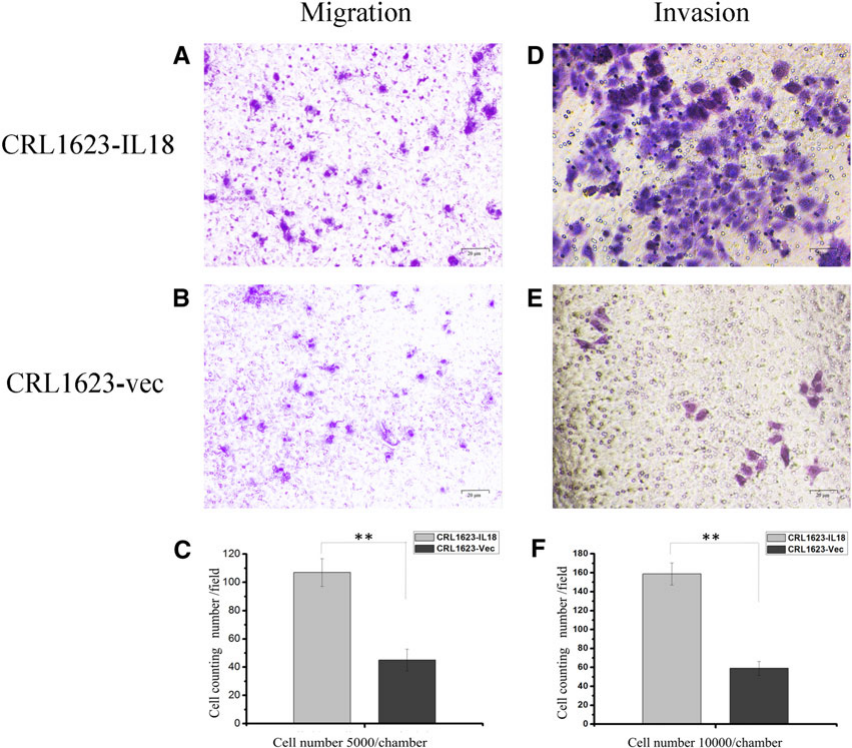
Effect of IL‐18 overexpression on promotion of TSCC cell migration and invasion (A,B ×400; D,E ×400). (A–C) After being cultured for 24 h in the Transwell upper chamber, TSCC cell migration capacity was upregulated in CRL1623‐IL‐18 compared with that of CRL1623‐vec cells (**P <* *0.01). (D–F) After being cultured for 48 h in the Transwell upper chamber with Matrigel (precoated) filters, tumor cell invasion capacity was upregulated in CRL1623‐IL‐18 compared with that of CRL1623‐vec cells (**P <* *0.01). The data were expressed as the mean ± SD and statistically analyzed using Student's *t* test in (C,F).

We then detected the migration and invasion capacity of CRL1623‐IL‐18 and CRL1623‐vec cells using the Transwell assay. As shown in Fig. 2, the tumor cell migration rate was higher in CRL1623‐IL‐18 than in CRL1623‐vec cells during the 24‐h time period (*P *<* *0.01; Fig. 2A–C). Moreover, tumor cell invasion rate was higher in CRL1623‐IL‐18 than in CRL1623‐vec cells during the 48‐h time period (*P *<* *0.01; Fig. 2D–F). These results suggest that the abilities of OSCC cell migration and invasion were increased.

### IL‐18 inhibition of cell viability, induction of cell apoptosis, and nude mouse xenograft growth

**Figure 3:**
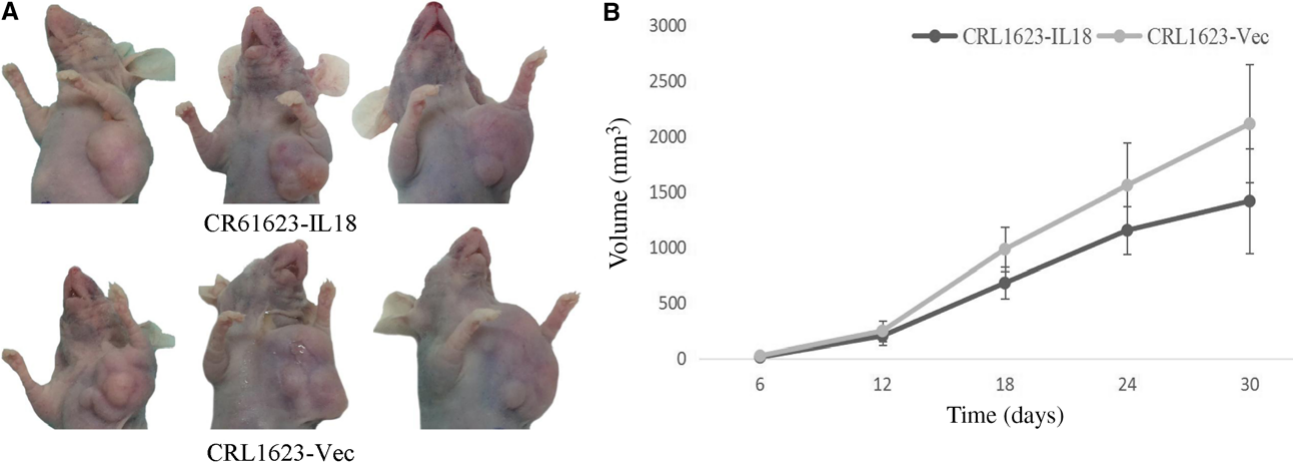
Effect of IL‐18 overexpression on inhibition of mouse xenograft growth. (A) Nude mouse TSCC cell xenograft model. (B) The growth curve of TSCC cell xenografts over 30 days. CRL1623‐IL‐18 cell‐injected nude mice had a slower xenografts growth, and the xenograft volume was also smaller than those of CRL1623‐vec cell‐injected mice (*P *<* *0.05). The data are expressed as the mean ± SD.

In a previous study, we showed that IL‐18 expression was higher in CRL1623‐IL‐18 cells than in CRL1623‐vec cells 9, 10 and that IL‐18 expression reduced OSCC cell viability, and increased apoptosis 9. After inoculation with tumor cell suspension, all the mice developed tumor burdens within 30 days (Fig. 3A). During the 30‐day period, tumor volumes increased in all of the mice; however, the growth speed of CRL1623‐IL‐18 xenografts was slower and the tumor xenograft volumes were smaller in size compared with those of the CRL1623‐vec‐injected mice (*P* < 0.05; Fig. 3B), thus indicating an inhibitory effect of IL‐18 on TSCC cells.

### IL‐18 regulation of expression of proteins in tumor xenografts

**Figure 4:**
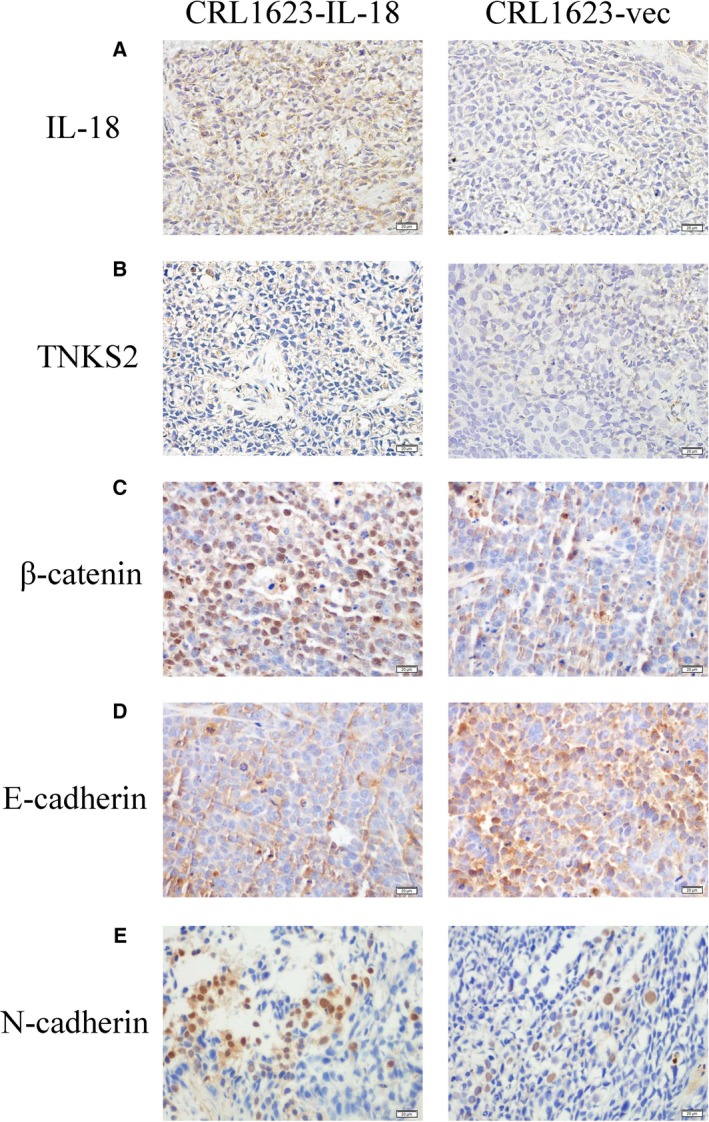
Expression of various proteins in TSCC cell xenografts (×400). (A,B,C,E) Levels of IL‐18, TNKS2, β‐catenin, and N‐cadherin were higher in CRL1623‐IL‐18 cell xenografts compared with those of CRL1623‐vec cell xenografts. (D) Expression of E‐cadherin was lower in CRL1623‐IL‐18 cell xenografts than that of CRL1623‐vec cell xenografts. Scale bars: 20 µm.

IL‐18 overexpression regulated expression of other proteins in nude mouse xenografts (Fig. 4). In particular, compared to the vector control, CRL1623‐IL‐18‐injected tumor xenografts expressed high levels of IL‐18 (*P *<* *0.01), β‐catenin (*P* = 0.028), TNKS2 (*P* = 0.045), and N‐cadherin (*P* = 0.068), but lower levels of E‐cadherin (*P* = 0.045), and thus the E/N ratio was changed (Table 4). In contrast to the data in OSCC tissue samples, IL‐18 overexpression was associated with levels of TNKS2 (*P* = 0.06) and β‐catenin (*P* = 0.04; Table 3). Moreover, β‐catenin expression was inversely associated with E‐cadherin (*P* = 0.23) but associated with N‐cadherin expression (*P* = 0.26).

**Table 4:** Expression of different proteins in TSCC xenografts. E/N, E‐cadherin*/*N‐cadherin ratio. Statistical analysis was by Student's *t* test

Protein	IOD		*P*
	CRL1623‐IL‐18	CRL1623‐vec	
IL‐18	0.0242 ± 0.0064	0.0086 ± 0.0067	0.002
TNKS2	0.0036 ± 0.0016	0.0019 ± 0.0009	0.045
β‐catenin	0.0040 ± 0.0016	0.0023 ± 0.0005	0.028
E‐cadherin	0.0033 ± 0.0023	0.0074 ± 0.0037	0.045
N‐cadherin	0.0024 ± 0.0008	0.0016 ± 0.0005	0.068
E/N	1.59 ± 1.33	5.01 ± 3.09	0.032

## Discussion

As a common malignancy in the head and neck region, OSCC can be effectively treated with targeted therapy to reduce patient mortality. Novel targets are even more effective in controlling tumor burdens in patients. Therefore, our current study assessed expression of IL‐18 and the corresponding proteins in OSCC tissue specimens and then investigated the role of IL‐18 overexpression in OSCC cells *in vitro* and in nude mouse xenografts.

Our data showed that levels of IL‐18 and E‐cadherin were reduced in OSCC, whereas β‐catenin, N‐cadherin, and TNKS2 proteins were upregulated in OSCC. The expression of IL‐18 and E‐cadherin was associated with tumor differentiation, whereas the expression of β‐catenin, N‐cadherin, and TNKS2 was associated with tumor de‐differentiation. Furthermore, although our *in vitro* data showed that IL‐18 promoted TSCC cell migration and invasion, our nude mouse assay revealed that IL‐18 suppressed growth of nude mouse xenografts. However, expression of other corresponding proteins was variable between the *ex vivo* and nude mouse xenografts for reasons that currently remain unclear. Thus, further study of IL‐18 in OSCC is needed to clarify its role in OSCC.

Previous studies demonstrated that IL‐18 could be a candidate for targeting therapy of human cancers since it possesses an antitumor effect due to recognition of NK cell receptors 33 or activates the p38 mitogen‐activated protein kinase signaling pathway 35, 36. Recently, IL‐18 was shown to be involved in therapy for melanoma 37, renal cell carcinoma 38, and lung cancer 39. In our previous study 40, we also found that IL‐18 could enhance expression and activation of caspase 3, 7, and 9 as well as IFN‐γ.

In our current study, we demonstrated how IL‐18 overexpression could suppress the growth of nude mouse OSCC cell xenografts, which corresponded to the results of MTT, annexin V/propidium iodide, and cell cycle assays in a previous study 11, showing IL‐18 inhibition in a time‐dependent manner especially after 48 h and inducing apoptosis of TSCC cells in an early stage. Moreover, IL‐18 expression and E‐cadherin expression were associated with OSCC differentiation, whereas N‐cadherin expression was associated with OSCC de‐differentiation, indicating that IL‐18 could inhibit tumor cell EMT. However, other studies gave opposite results; namely, IL‐18 was more likely to promote tumor cell EMT. For example, Kang *et al*. 41 discovered that IL‐18 expression was able to induce gastric cancer cell immune evasion by upregulation of CD44 and vascular endothelial growth factor in nude mouse gastric cancer xenografts, while Li *et al*. 42 reported that IL‐18 expression was upregulated after activation of the phosphatidylinositol 3‐kinase/serine–threonine kinase (Akt) signaling pathway in promotion of breast cancer metastasis.

To define the effect of IL‐18 on OSCC cells, we assessed the migration and invasion capacity of TSCC cells after IL‐18 overexpression using the Transwell assay *in vitro* and analyzed expression of IL‐18‐associated proteins in TSCC tissues using immunohistochemistry. Our Transwell assay data showed that TSCC cells overexpressing IL‐18 had a stronger ability to migrate and invade *in vitro* (Fig. 2), while IL‐18 expression was associated with TNKS2 and N‐cadherin expression, but inversely associated with E‐cadherin expression in TSCC tissues (Fig. 4 and Table 4), indicating that IL‐18 was able to induce TSCC cell migration and invasion and expression of EMT‐related proteins, such as N‐cadherin. It seemed like a discrepant effect of IL‐18 on cell motility. We suppose that IL‐18 may act at different sites in a dose‐dependent manner and the sites play a reverse role. However, the concrete reason for this discrepancy is unknown and further study is needed to investigate it using different cell lines.

Multiple studies have confirmed that activation of β‐catenin is closely related to the occurrence of EMT 43, 44, 45. Our current study demonstrated that β‐catenin expression was upregulated in OSCC tissue samples (compared with that of normal oral mucosae) and associated with tumor de‐differentiation (Fig. 2C and Table 1). In the TSCC cell xenograft model, IL‐18 overexpression manifested higher levels of β‐catenin than in the control group (Fig. 4C and Table 4). Interestingly, the expression tendency of TNKS2 was similar to β‐catenin in both OSCC and TSCC xenograft specimens, and IL‐18 overexpression was positively correlated with TNKS2 and β‐catenin expression (Table 3), demonstrating TNKS2 was consistent with β‐catenin and promoted cell migration and invasion 28, 46. Therefore, we supposed that IL‐18 overexpression could activate β‐catenin signaling by stimulation of TNKS2 expression, resulting in the switch between E‐cadherin and N‐cadherin expression, namely increase in N‐cadherin expression but decrease in E‐cadherin expression to reverse the ratio of E‐cadherin to N‐cadherin and induced tumor cell EMT.

## Conclusions

In conclusion, our current study, together with our previous data 9, 11, demonstrated that IL‐18 had double effects on OSCC—i.e. inhibition of tumor growth by induction of the tumor cell apoptosis pathway through glycogen synthase kinase 3β 9, 11 and promotion tumor cell EMT by activation of TNKS2 via the Wnt/β‐catenin signal pathway *in vitro*, and could therefore play a role in tumor metastasis. However, if IL‐18 has the capacity to promote metastasis *in vivo*, despite a reduction in tumor size, and the molecular mechanisms by which IL‐18 induces tumor cell EMT in OSCC *in vitro* need further investigation.

## Author contributions

WL and YL conceived and designed this study. YL, ZX, JL, SB, and CD performed the experiments. YL, SB, and CD analyzed the data. YL, ZX, and JL prepared the manuscript. WL and YL revised and provided critical discussion of the manuscript. WL provided the funding support in the study.

## Conflict of interest

The authors declare no conflict of interest.
